# The news from RBMT

**DOI:** 10.47626/1679-4435-2020-182

**Published:** 2020-12-11

**Authors:** Andrea Franco Amoras Magalhães

**Affiliations:** Editor-in-Chief, Revista Brasileira de Medicina do Trabalho

We proudly present the second issue of volume 18 of the *Brazilian Journal of Occupational Medicine* (*Revista Brasileira de Medicina do Trabalho* - RBMT). In this issue, we introduce some changes that aim to help the journal to overcome the challenges of a scientific publication, including, but not limited to, improving the quality of the journal and expanding the list of collaborators.

Thus, we are proud to inform that we have increased the number of reviewers registered in the system, which will allow us to reduce the authors’ waiting time for approval of submitted articles. In parallel with that, we have observed a significant increase in the number of articles submitted for publication, a phenomenon that reflects the journal’s growing prestige.

Furthermore, in order to meet the demands for improvement in journal publishing processes imposed by indexing bodies, we have decided to change the publisher responsible for producing the journal. Our new partner is Scientific Linguagem, a renowned Brazilian publisher that has been operating in the scientific publishing market for over 20 years, supporting both authors and editors. We would also like to highlight that the RBMT website, designed by GN1, has been renovated, having now a new layout that is modern, clean, and more comfortable to navigate.

All of those actions are, in the view of the editors, required to improve the journal, and they take time to be implemented. However, we believe that we are on the right path and that we will soon be reaping the rewards of those decisions.

We are continuously improving to attract more authors, from both national and international audiences, to increase the journal’s visibility, and to achieve the goals proposed by the current administration.

